# Sense of agency is related to gamma band coupling in an inferior parietal-preSMA circuitry

**DOI:** 10.3389/fnhum.2014.00510

**Published:** 2014-07-16

**Authors:** Anina Ritterband-Rosenbaum, Jens B. Nielsen, Mark S. Christensen

**Affiliations:** ^1^Department of Nutrition, Exercise and Sports, University of CopenhagenCopenhagen, Denmark; ^2^Department of Neuroscience and Pharmacology, University of CopenhagenCopenhagen, Denmark; ^3^Cognitive Neuroscience Research Unit, Danish Neuroscience Center, Aarhus UniversityAarhus, Denmark

**Keywords:** sense of agency (SoA), supplementary motor area (SMA), right inferior parietal cortex (IPC), dynamic causal model (DCM), γ-activity in SMA-IPC network

## Abstract

In the present study we tested whether sense of agency (SoA) is reflected by changes in coupling between right medio-frontal/supplementary motor area (SMA) and inferior parietal cortex (IPC). Twelve healthy adult volunteers participated in the study. They performed a variation of a line-drawing task (Nielsen, 1963; Fourneret and Jeannerod, 1998), in which they moved a cursor on a digital tablet with their right hand without seeing the hand. Visual feedback displayed on a computer monitor was either in correspondence with or deviated from the actual movement. This made participants uncertain as to the agent of the movement and they reported SoA in approximately 50% of trials when the movement was computer-generated. We tested whether IPC-preSMA coupling was associated with SoA, using dynamic causal modeling (DCM) for induced responses (Chen et al., 2008; Herz et al., 2012). Nine different DCMs were constructed for the early and late phases of the task, respectively. All models included two regions: a superior medial gyrus (preSMA) region and a right supramarginal gyrus (IPC) region. Bayesian models selection (Stephan et al., 2009) favored a model with input to IPC and modulation of the forward connection to SMA in the late task phase, and a model with input to preSMA and modulation of the backward connection was favored for the early task phase. The analysis shows that IPC source activity in the 50–60 Hz range modulated preSMA source activity in the 40–70 Hz range in the presence of SoA compared with no SoA in the late task phase, but the test of the early task phase did not reveal any differences between presence and absence of SoA. We show that SoA is associated with a directionally specific between frequencies coupling from IPC to preSMA in the higher gamma (ɣ) band in the late task phase. This suggests that SoA is a retrospective perception, which is highly dependent on interpretation of the outcome of the performed action.

## Introduction

When we reach for a cup of coffee we usually feel that we are in control of what we are doing and that we are the agent of the movement. Current research suggests that the sense of agency (SoA) occurs when the sensory consequences (usually in the form of proprioceptive and visual feedback) of the movement correspond to the original intention and plan of the movement, i.e., the comparator model (Gallagher, 2000; Wegner, 2004; Engbert et al., 2008). The comparator model fits within the experimental framework of feedback manipulations, in which (typical) visual feedback is distorted in such a way that there is a mismatch between visual and proprioceptive feedback, and thereby also a mismatch between the intended action outcome and the visual feedback.

The most common way to study the SoA is to expose participants to a situation of ambiguity regarding self-produced movement. This may be done by manipulating the visual feedback that a participant receives regarding performance of a simple hand or arm movement. Nielsen (1963) introduced the first version of this experimental design (known as the Alien Hand paradigm). He was not interested in SoA *per se*, but rather the feeling of volition, which is essential for SoA (Nielsen, 1963). These types of manipulations also allow participants to focus on the judgmental task of determining whether they themselves or an external agent performed the action (Farrer et al., 2003a). Such tasks have led to the notion of a “who”-system (Georgieff and Jeannerod, 1998), which is used in the process of determining “who” is the agent. Several later studies have adapted the paradigm to investigate intentional actions and the neural mechanisms underlying SoA (e.g., Fourneret and Jeannerod, 1998; Farrer and Frith, 2002). However, recent studies suggest that unexpected outcomes of actions are associated with high sense of control if the action leading to the response is compatibly primed (Chambon et al., 2013; Sidarus et al., 2013), suggesting that SoA depends on prospective forms of knowledge relating to action selection processes, and independent of action outcome.

However, the neural circuitry responsible for the experience of agency has not been fully clarified in terms of how brain regions interact and the temporal aspect of activities in specific cortical structures. Several studies have implied that areas in the inferior parietal cortex (IPC) and areas in the supplementary motor area (SMA) are involved in the formation of intentions prior to the movement and evaluation of action outcomes (Sirigu et al., 1999, 2004; Leube et al., 2003; Farrer et al., 2008; Desmurget et al., 2009). Patients with lesions of the posterior parietal cortex (PPC) more often mistake whether they are or an experimenter is responsible for a movement shown on a video screen (Sirigu et al., 1999) and they become aware of their decision with a significant delay compared to healthy participants (Sirigu et al., 2004). Electrical stimulation of the right inferior parietal lobe (IPL) may also make participants falsely believe that they moved or intended to move (Desmurget and Sirigu, 2009), while stimulation of the SMA has been reported to produce an urge to move (Fried et al., 1991). Imaging studies have demonstrated activation in the preSMA and in the right angular gyrus (part of the IPC) when participants experience a discrepancy between intended and observed movements (Farrer and Frith, 2002; Farrer et al., 2008; Yomogida et al., 2010; Nahab et al., 2011; Chambon et al., 2013), which also shows parametric modulations when deviations are increased gradually (Farrer et al., 2003a). Interruption of preSMA by transcranial magnetic stimulation demonstrated disruption of agency (Moore et al., 2010). While these studies provide evidence of the involvement of the respective areas in the generation of the subjective SoA, they reveal nothing about the functional or effective connectivity or the temporal aspect of neural communication in the parietal-SMA network involved in SoA.

In order to elucidate these issues we conducted the present EEG-study of the time course of coupled activity in the right IPC-preSMA network in relation to SoA. The analysis of the study was explorative. However, we hypothesized a modulation of activity between the selected target regions depending on behavior of the participant and the reflective task the participants were exposed to in a modified version of the Alien-Hand Paradigm (Nielsen, 1963). The connectivity between the two regions of interest was disclosed using a dynamic causal model (DCM) (Chen et al., 2008) for induced responses, in which a Bayesian Model Selection (BMS) was used to select the DCM, which explains the activity in right IPC and preSMA and how these are coupled best. The DCM describes how the neural activity, in terms of oscillatory power of one brain region, modulates the activity, again in terms of oscillatory power, of another region. This was investigated in relation to a motor task in which participants were asked to judge whether they themselves were responsible for a cursor movement presented on a computer screen, or whether a computer was responsible. Previous studies have shown that conscious perception of visual input is associated with coupling of neural activity across cortical areas in the ɣ-band frequency range (Rodriguez et al., 1999; Engel et al., 2001; Palva et al., 2005; Melloni et al., 2007; Siegel et al., 2012). Furthermore, investigations of EEG activity in relation to SoA have focused on modulation of event related potentials (Balconi and Crivelli, 2009; Gentsch et al., 2012), showing increased N1 components for externally-generated visual feedback and increased ERP components around 100 ms for delayed visual feedback respectively. However, to our knowledge no studies have looked at oscillatory coupling in relation to SoA We hypothesized that a network with information flow from preSMA-IPC would be favored in the initial phase of the movement, indicating that formation of intention of the action is formed in frontal regions and fed to parietal regions for later comparison between intended and actual movement outcome. Hence, opposite direction of information flow would be favored in the late phase of the task.

## Materials and methods

We included 12 right-handed, healthy adults (10 men/2 women) ranging from 22–32 years (average: 26.4 ± 2.8 years). None of the participants had any history of neurological or psychiatric disorder. Two male participants were excluded from the analysis after initial inspection of the data files revealed that they displayed very odd subjective reports of agency, i.e., reporting YES (or NO) in more than 80% of all trials. Ten participants were therefore included in the analysis. All participants were given written and oral information and all signed a consent form before the start of the experiment. The experiment was carried out according to the Helsinki-declaration and with approval from the local ethics committee of the Capital Region of Copenhagen (protocol number: H-B-2009-17).

### Experimental procedure

**Figure 1:**
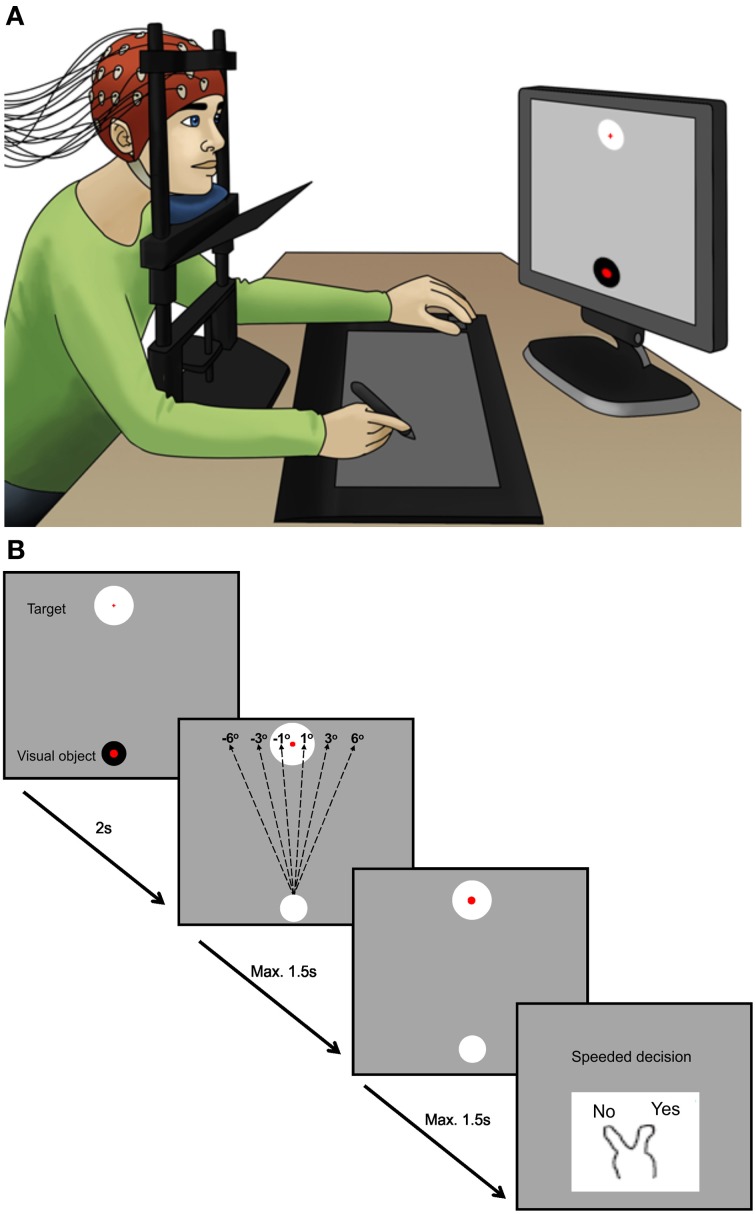
**The experimental design. (A)** Displays the position of the participants during the experiment. The plate below the chin blocks the view of the hands but participants can follow the consequence of the action on the screen in front of them. **(B)** Illustrates what is occurring on the computer screen. During the experiment the cursor was randomly manipulated from the beginning of the movement by different angles of (−6, −3, −1, 1, 3, 6°). Negative angles refer to manipulation to the left side of the target. Participants had max. 1.5 s to perform the movement, but were instructed to move fast. When reaching the level of the target, the visual object disappeared and participants had to report as fast as possible (within 1.5 s) whether or not they felt they were the agents of the observed movement by a key press of the index or middle finger of the left hand. After key press, participants had 2 s to place the pen on top of the visual object and be ready for the next trial. During the experiment there were no lines visible on the display screen.

The experimental paradigm was adapted from Ritterband-Rosenbaum et al. (2011) and aimed to cause ambiguity as to whether the participant or a computer was responsible for moving a cursor on a computer screen (Ritterband-Rosenbaum et al., 2011). The participants were seated comfortably in a chair with their heads in chin-rests 55 cm away from a computer screen. Vision of participants' own hands was blocked during the entire experiment (see Figure 1). Participants were instructed to make a fast (within 1.5 s) straight movement in the sagittal plane away from the center of their body by moving a cursor with a pen on a pen-tablet (Wacom, Intuos 3, Krefeld, Germany). The task was presented in a custom made Matlab (The MathWorks, Natics, MA, USA) program. A cue appeared on the screen indicating when participants were to start the movement. Participants had to move the cursor from the starting position at the bottom of the screen to the target at the top of the screen. When reaching the vertical level of the target, the cursor disappeared and participants had to report as fast as possible (within 1.5 s) whether they felt themselves as the agents of the observed movement. This was done by a key press of either the index finger (“yes, it is me”) or middle finger (“no, it is not me”) of the left hand. After the key press the participants had 2 s to place the pen to be ready for next trial. Each trial lasted 7 s. All participants performed 2 blocks of 200 trials (25 min) interrupted by a small break of 2–5 min to assure participants were attentive.

Three types of experimental trial types were introduced: Computer manipulated movements, Self-generated movements (trials with no interference of the computer on the observed movement), and Pause trials. The computer manipulated movements consisted of trials where the visual feedback was manipulated 1, 3, 6 (right), −1, −3, and −6 (left) degrees away from target (Figure 1), i.e., in parametrical fashion as done previously in other studies (Fourneret and Jeannerod, 1998; Farrer et al., 2003a,b). The 3/−3° manipulations were presented 80 times, the other angles 40 times. The self-generated movements and Pause trials were presented 40 times each. The Pause trials initially displayed the instruction “Pause” on the screen, after which the cursor moved to the target while participants did not move the hand. The trials were presented in a random order, though the order was the same for all participants. The computer-manipulations were induced from the beginning of the movement and continued in a straight line to the predefined position at the same level as the target. The dimensions of the tablet were 310 × 238 mm and the dimensions of the screen were 380 × 303 mm (with a resolution of 1280 × 1024 pixels). Moving 1 cm on the tablet corresponded to 1.2 cm horizontally and 1.3 cm vertically on the screen. The cursor was placed centrally 20% from the bottom of the screen with a diameter of 0.2 cm. To reach the target, which was located centrally 20% from the top of the screen, participants had to move approximately 15 cm on the tablet, which could easily be done without moving the full body.

We aimed to find movement deviation, which corresponded to 50/50% self-reported agency distribution. Pilot experiments indicated that this ratio was obtained at −3/3° deviations; therefore we exposed the participants to a higher number of −3/3° trials, but in the actual series of experiments the angle of deviation at which this ratio was found differed between participants, and as a consequence data from all angle deviations were pooled across all participants. Trials where the time of the answer was longer than the allowed response time (1.5 s) were excluded from the analysis (1.8% of all trials were excluded corresponding to less than 5.3% from 1 of the participants, less than 2.8% from 9 of the participants, and 0% from 2 participants). A total of 2972 trials with a ratio of roughly 50/50 for agency/no agency reporting were used in the EEG analysis (see also section EEG data analysis for further description). For individual participants the ratio varied from 13 to 72% for agency attribution with an average ratio for attributing the movement to one self of 42%.

### Data acquisition

Continuous EEG was recorded from 64 channels (ActiveTwo, BioSemi, Amsterdam, The Netherlands) using acquisition software ActiView (version 6.05). Active electrodes were mounted in a headcap (headcap BioSemi, The Netherlands). Off-set was kept below 25 microV. Recordings were set to AC and 1 Hz high-pass filtering applied. Sampling rate was 2048 Hz. Markers indicating onset of movement, end of movement, and key-press when reporting experience of agency were co-registered with the EEG.

### EEG data analysis

All data were analyzed offline using Matlab R2010a (MathWorks, MA, USA), with the toolbox EEGLab v9.0.4.4b (Swartz Center for Computational Neuroscience; http://sccn.ucsd.edu/eeglab/), and the toolbox Statistical Parametric Mapping (SPM8).

Files were imported to EEGlab, resampled to 256 Hz in order to reduce computation time, re-referenced to average reference. Then 1 Hz high-pass and 80 Hz low-pass filters were applied. Independent Component Analysis (ICA) was applied using the runica algorithm. ICA components reflecting eye-blinks and lateral eye-movements were identified by visual inspection and subsequently removed from the data. If noise components that were visible as noise across the whole scalp image were identified, these were also removed from the data. The EEG data without eye movement and common noise artifacts were exported from EEGlab to BDF-format files.

The new BDF files were imported into SPM8. Data were epoched from −500 to +1000 ms with respect to movement onset for each trial. The epochs were sorted into AgencyYES and AgencyNo trials. All epochs were visually inspected and in trials with spikes or similar artifacts were declared as “BAD” and left out of further analysis. On average 8% of all epochs were excluded.

The epoched data were then taken into an initial source image analysis using empirical Bayes Methods as implemented in SPM8. EEG data were co-registered with a template T1-weighted magnetic resonance image, and a forward model was constructed using a Boundary Element Model. The forward model was inverted using the multiple sparse priors as hyper priors. Data were limited to a time window from 0 ms to +800 ms with respect to movement onset. Furthermore, the data were limited to frequencies between 4 and 80 Hz. Images of the reconstructed sources were separated into an early (0–400 ms) and late (400–800 ms) task phases, and divided into delta (4–7 Hz), alpha (8–14 Hz), beta (15–30 Hz), low ɣ (31–50 Hz) and high ɣ (51–80 Hz) frequency ranges, based on textbook frequency separations which are supposed to reflect different functional properties related to alertness, motor control, attention, conscious thoughts, etc., and into AgencyYES and AgencyNO conditions. Studies suggest that conscious perception depends on transient synchronized activity at frequencies around 30–60 Hz; We therefore found it important to look at different frequency bands and to further separate the ɣ-band into low and high ranges (Rodriguez et al., 1999; Engel et al., 2001; Palva et al., 2005; Melloni et al., 2007). We chose to separate the early and late task phases as we believed that the two time periods are related to different events of the task presented. The early phase governs the movement as such, whereas the late phase represents evaluation of the movement and therefore a different modulatory activity. These images, reconstructed for each participant, were taken into a second level 3-way ANOVA analysis. An *F*-test was made across all conditions, i.e., the mean of all conditions. Results from this source analysis gave rise to an image of areas that were used to guide the subsequent Dynamic Causal Model (DCM) analysis. Furthermore, we performed a test of the main effect of agency on the source analysis images which was also used to guide the DCM analysis and tests of the main effects of time and frequency.

DCM for induced responses (Chen et al., 2008) was used in order to assess within-frequency and between-frequency coupling between medial frontal and right inferior parietal regions. We were particularly interested in the differences in coupling between the AgencyYES and AgencyNO trials. We therefore tested whether or not ascribing the visually perceived action to one-self would be reflected in different coupling patterns in a network of regions involved in this action.

**Figure 2:**
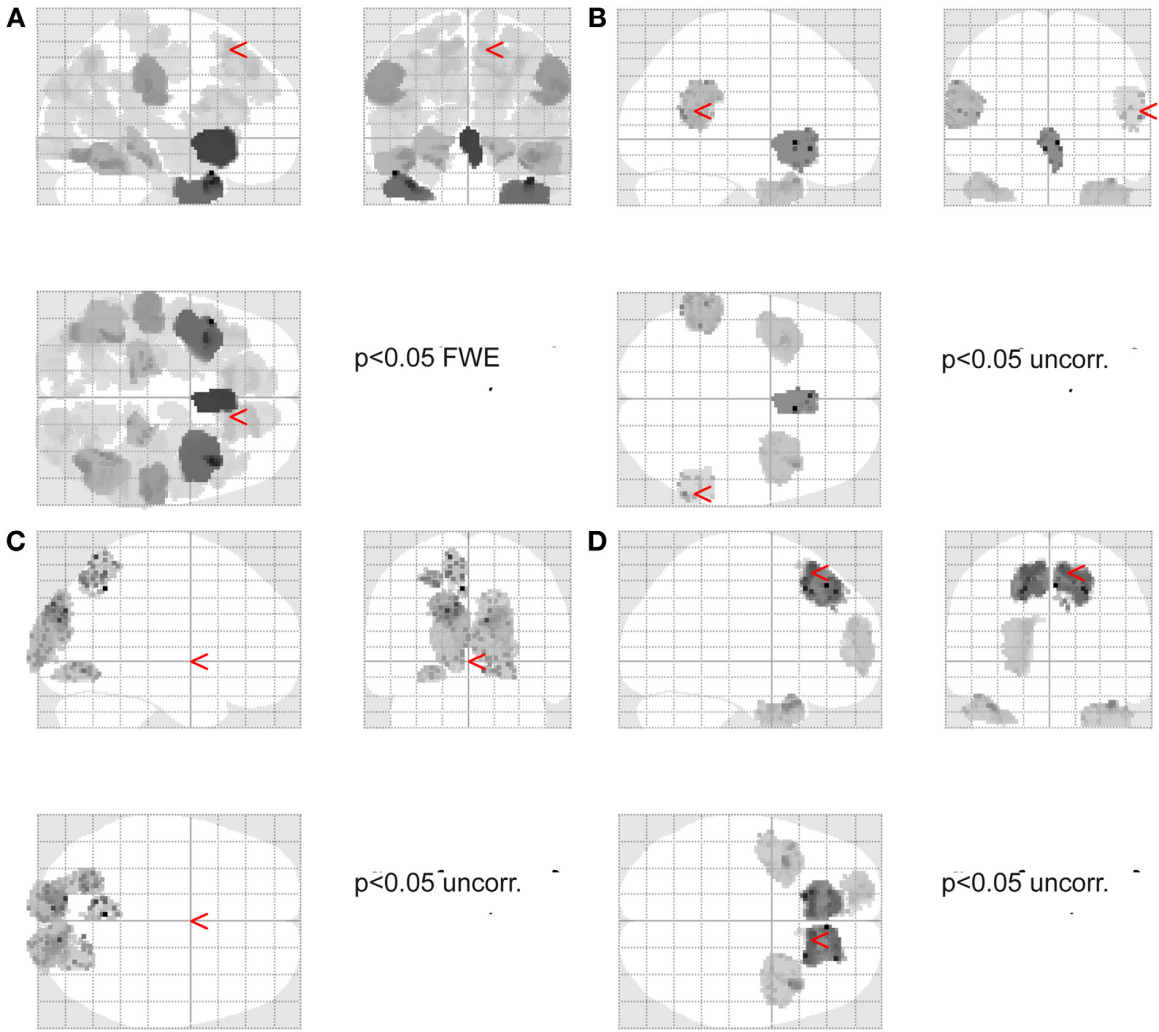
**Source localization. (A)** Shows the main effect across all conditions (*p* < 0.05 FWE corrected for multiple comparisons). Red arrow points to the location used for the DCM analysis for the preSMA source. **(B)** Shows the main effect of agency (*p* < 0.05 uncorrected threshold). Red arrows point to the location used for the DCM analysis for the IPC source. **(C)** Shows the main effect of time (i.e., early vs. late) (*p* < 0.05 uncorrected threshold) (arrow in MNI 0,0,0). **(D)** Shows the main effect of frequency (*p* < 0.05 uncorrected threshold). Red arrow points to the location used for the DCM analysis for the preSMA source.

We selected two regions that have been implicated in motor tasks that include judgments of agency. These were a medial frontal region and right IPC. Two loci, which were used as prior for the DCM source reconstruction procedure, were chosen based on the clusters found in the imaging source analyses (Figure 2) described above. These were: right fronto-medial region (preSMA, superior medial gyrus, MNI coordinate: 12, 26, 56) based on the source analysis of the mean across all conditions in the above described ANOVA, and right inferior parietal region (supramarginal gyrus, IPC(PGa), MNI coordinate: 60, −50, 18) based on the analysis of the positive main effect of Agency, i.e., Agency YES > Agency NO. These two regions were used in the subsequent DCM analyses. Furthermore, two different sets of models were constructed, one corresponding to the early part of the task (1–400 ms), and one corresponding to the late part of the task (400–800 ms), covering the time when participants evaluate their movement.

Nine different DCM were constructed from the data from the early task phase (1–400 ms time window) and nine DCMs from the late task phase (400–800 ms). All models included the right preSMA (MNI: 12, 36, 56) and right IPC (MNI: 60, −50, 18) regions. Two types of effects were constructed: the AgencyYES and AgencyNO trials, i.e., SoA condition. These effects were allowed to enter either one or both of the regions; the effects could either influence the coupling from the frontal to the parietal region, the coupling from the parietal to the frontal, or both couplings at the same time. In all models information can “flow” between both regions, but it is the information about SoA that influences the models differently. In models 1–3, SoA can influence both connections between the regions; in models 4–6 it is only information flowing from IPC to preSMA that is influenced by SoA, and in models 7–9 it is only information flowing from preSMA to IPC that is influenced by SoA. Models 1, 4, and 7 are similar with respect to where information about SoA should enter the models, in these cases into both IPC and preSMA. Models 2, 5, and 8 are similar in the sense that information enters preSMA, and in Models 3, 6, and 9 information enters IPC. If any of Models 1–3 are favored by a Bayesian Model Selection (BMS) analyses it indicates that SoA is a process that requires that information between IPC and preSMA has to be reiterated between the two regions. If any of Models 4–6 are favored in a BMS it indicates that intentional information about the predicted consequences of the action, formed in preSMA, is modulated by SoA, and if any of Models 7–9 are favored by a BMS it indicates that actual sensory consequences, or deviations between expected and actual consequences, computed in IPC are modulated by SoA. If models 1, 4, or 7 are favored it indicates that SoA is “generated” simultaneously in IPC and preSMA, which would mean that any distinction of whether SoA depends mainly on information about the intention of the movement or depends on the outcome of the comparison between expected and actual feedback remains unresolved.

For this DCM for induced responses we chose a non-linear coupling, i.e., allowing between-frequency coupling in the range between 4–80 Hz, because this allows modeling both within-frequency coupling and between frequency coupling. This choice was made because “Agency” as a phenomenon incorporates aspects of motor control as well as aspects of conscious self-recognition, and these behaviors are not necessarily associated with EEG oscillations at the same frequencies. These combinations gave rise to the nine different DCMs displayed in **Figure 4**, which then was constructed for the two different task phases (early and late).

In order to determine which of the two times nine models explained the data best, we conducted two separate fixed effect BMS analyses, one for the early task phase (1–400 ms) and one for the late task phase (400–800 ms).

The models that explained the data best selected from the two BMS of the early task phase and late task phase respectively were used for subsequent comparisons. Here the coupling between the frontal and parietal regions was tested using paired *t*-tests. The *t*-tests compared the frequency-frequency images of the effect of trials on the coupling derived from the respective model.

### Behavioral data analysis

Only data from trials with manipulated angles were used for analysis. Group averages were done after separating data depending on the experience of agency for the different kinematical results. Each trial contains X_pen_, Y_pen_ coordinates for each individual movement produced by the pen on the tablet, and X_cursor_, Y_cursor_ coordinates for the trajectory of the cursor on the screen. Each complete set of coordinates was normalized to the size of the pen-tablet and used for further calculations of the kinematic:

1. Movement time (ms): refers to the time to complete the movement. Movement time is calculated by the start of the movement to the final point on the screen where the visual object reaches the vertical level of the target.
2. Hit distance (mm): corresponds to the distance between the end position X_pen_ and X_target_ center of the target. Negative and positive values indicate hit distances on the left side and right side of the target, respectively.
3. Line curvature (mm^−1^): indicates how curved the actual movement is. It was based on a calculation of the relative distance between the produced movement and the shortest distance to the target. The curvature measure for this purpose is the accumulated local curvature for the entire movement. A lower score represents a more direct movement toward the target. It is calculated by the formula.
  $$\text{C}=\;\frac{\text{x}\prime \text{y}\prime \prime -\text{y}\prime \text{ x}\prime \prime }{{\left(\text{x}\prime^{2}+\text{y}\prime^{2}\right)}^{3/2}}$$
4. Drift (mm): measures the difference between the movement of the pen on the tablet and the observed cursor movement trajectory on the computer screen. Small values indicate good correspondence between the produced and the observed movement. It was calculated as the Eucledian distance between the X_pen_,Y_pen_ and the X_object_,Y_object_
  $$\text{drift =}\sqrt{{\left({\text{x}}_{\text{pen}}-{\text{x}}_{\text{screen}}\right)}^{2}+{\left({\text{y}}_{\text{pen}}-{\text{y}}_{\text{screen}}\right)}^{2}}$$
5. Answer time (ms): indicates the time from the end of the movement until participants pressed a button to indicate whether they experienced agency or not.

Paired *T*-tests for the behavioral data were used and the alpha level set at 0.05. For non-normally distributed data a Mann-Whitney Rank Sum Test was applied.

## Results

### Behavioral findings

Table 1 reports the kinematics of the performed movement in relation to the subjective experience of agency. The movement time (Mann-Whitney Rank Sum: 49.0, *p* = 0.97), the line curvature (Mann-Whitney Rank Sum: 47.0, *p* = 0.583), the hit distance (Mann-Whitney Rank Sum: 49.0, *p* = 0.970), the drift (Mann-Whitney Rank Sum: 37.0, *p* = 0.345) and the answer time (*t* = −0.058, *p* = 0.477) were all very similar whether the participants experienced agency or not.

**Table 1:** **Kinematic for all deviations divided into the two categories of subjective reporting**.

	**Yes**	**No**
Movement time (ms)	332 ± 49	327 ± 52
Hit distance (mm)	−24.2 ± 37.4	−25.4 ± 37.2
Line curvature (mm^−1^)	0.033 ± 0.008	0.032 ± 0.007
Drift (mm)	43.8 ± 26.9	51.2 ± 27.5
Answer time (ms)	492 ± 119	489 ± 132

Table 1 provides information about group averages of kinematic results separating data into the subjective reporting. Inter-participant variance is given by 1 *SD*.

### Source localization

The initial image source localization analysis demonstrated significant sources (*F*-test, across all conditions, voxel threshold *p* < 0.05 Family Wise Error (FWE) corrected for multiple comparisons using Gaussian random field theory, limited to cortical areas associated with gray matter as defined by the SPM anatomy toolbox v1.8) in the right inferior temporal gyrus, right superior parietal lobule (angular gyrus, superior occipital gyrus, middle occipital gyrus, precuneus), left superior parietal lobule (angular gyrus, middle occipital gyrus), bilateral IPC (supramarginal gyrus), right inferior and medial temporal gyrus, left inferior occipital gyrus, bilateral inferior frontal gyrus, left inferior temporal gyrus and temporal pole, bilateral superior medial and superior frontal gyrus (see Figure 2A).

#### Exploratory source localization analyses

The main effect of agency showed significant differences in source strength in bilateral IPC (supramarginal gyrus) albeit at a lenient (*p* < 0.05 uncorrected) threshold (see Figure 2B) which was used for the subsequent DCM analysis.

The main effect of task phase (early vs. late) revealed significant albeit at a lenient threshold (*p* < 0.05 uncorrected) source differences in early visual areas and along the dorsal stream (Figure 2C).

The main effect of frequency revealed significant albeit at a lenient threshold (*p* < 0.05 uncorrected) sources in frontal areas, including the preSMA region, which was used for the subsequent DCM analysis.

Because DCM for EEG incorporates a generative model of the sources that are modeled, these initial source localization analyses are not necessary for the specification of the models. The statistics underlying the sources are not crucial for the specification of the DCM, since the DCM tests specific hypotheses concerning the sources incorporated in the model, and not an unspecific hypothesis concerning any combination of sources in the data. Therefore, these source analyses serve only as guidelines for the loci used in the DCM analyses. As part of the DCM for induced responses, we also employed a step that optimizes source localization. This optimization is based on the initial loci given, but allow for deviations away from the exact loci. The above mentioned source localization analyses of the three main effects serve only as an exploratory test for guidance. We based the IPC and preSMA loci for the DCM on the initial explorative source analyses. However, values based on previous studies would be an alternative, which would serve the same purpose.

### Dynamic causal model fit

**Figure 3:**
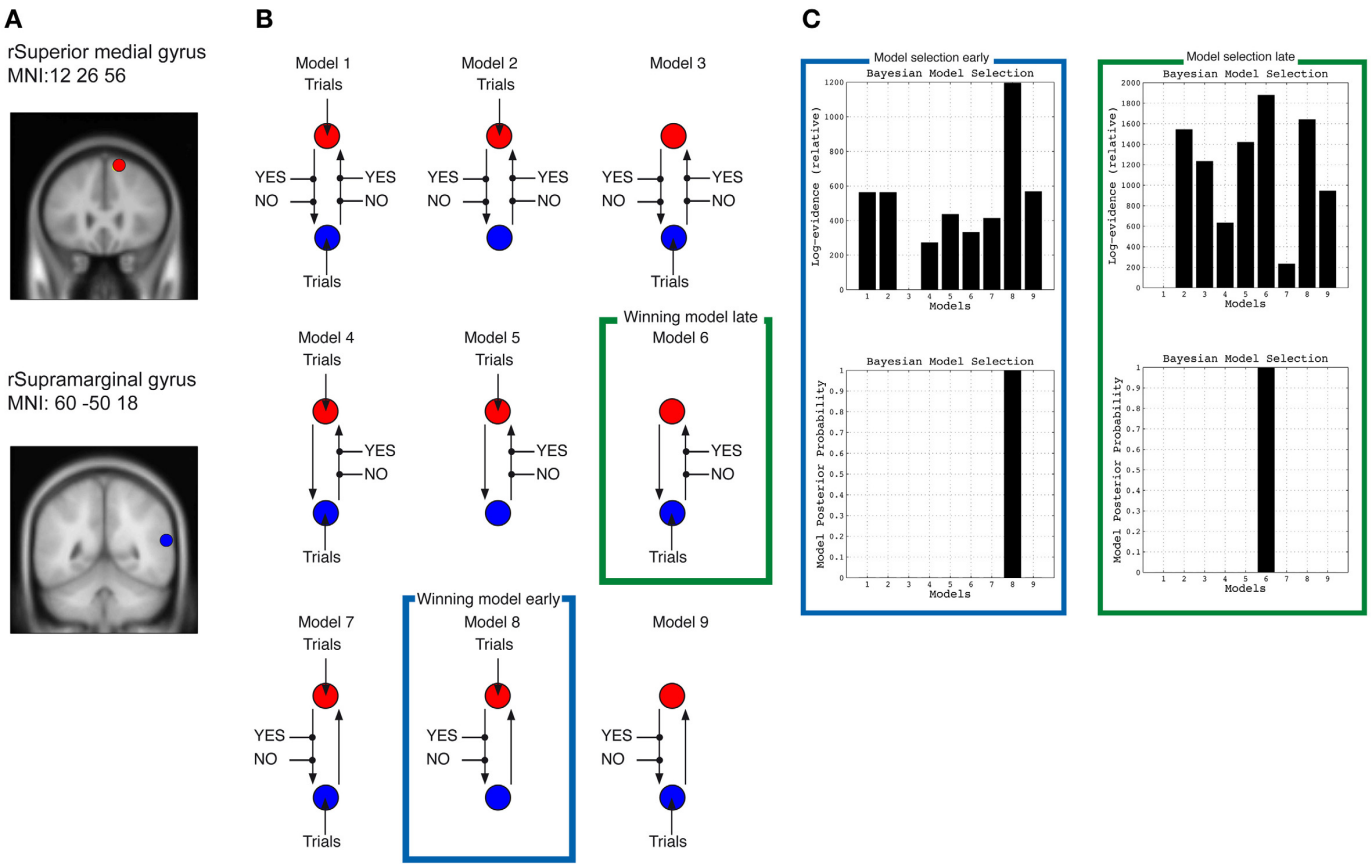
**Dynamic causal models. (A)** Shows location of the two sources used for the DCM analyses in right medial frontal gyrus ~preSMA and right supramarginal gyrus ~IPC. **(B)** Shows the 9 different DCMs, and **(C)** the results of the Bayesian model selection for the early phase (marked in green square) and late phase (marked in blue square) of the movement (400–800 ms).

The nine models (Figure 3), as described in the methods sections, were constructed for all 10 participants and underwent model inversion in SPM8 for the early and late task phase separately. For the early task phase data, all model inversions revealed models that showed time-frequency plots reflecting a simplified version of the actual data (see Supplementary Figure S1, which compares data from the two sources with the predictions derived from the models). For the late task phase, model inversions from one participant resulted in nine models without any dynamics, and hence the participant's models were not included in the subsequent BMS for the late phase.

### Bayesian model selection

The BMS revealed that Model 8 was the winning model for the early task phase, whereas Model 6 was the model that fitted the data best for the late task phase (Figure 3). Model 8 for the early task phase is the model in which information about SoA is fed into preSMA, and where SoA modulates the connection from preSMA to IPC. Model 6 for the late task phase is the model in which information about SoA is feed into IPC, and where SoA modulates the connection from IPC to preSMA.

### Dynamic causal model of induced responses

**Figure 4:**
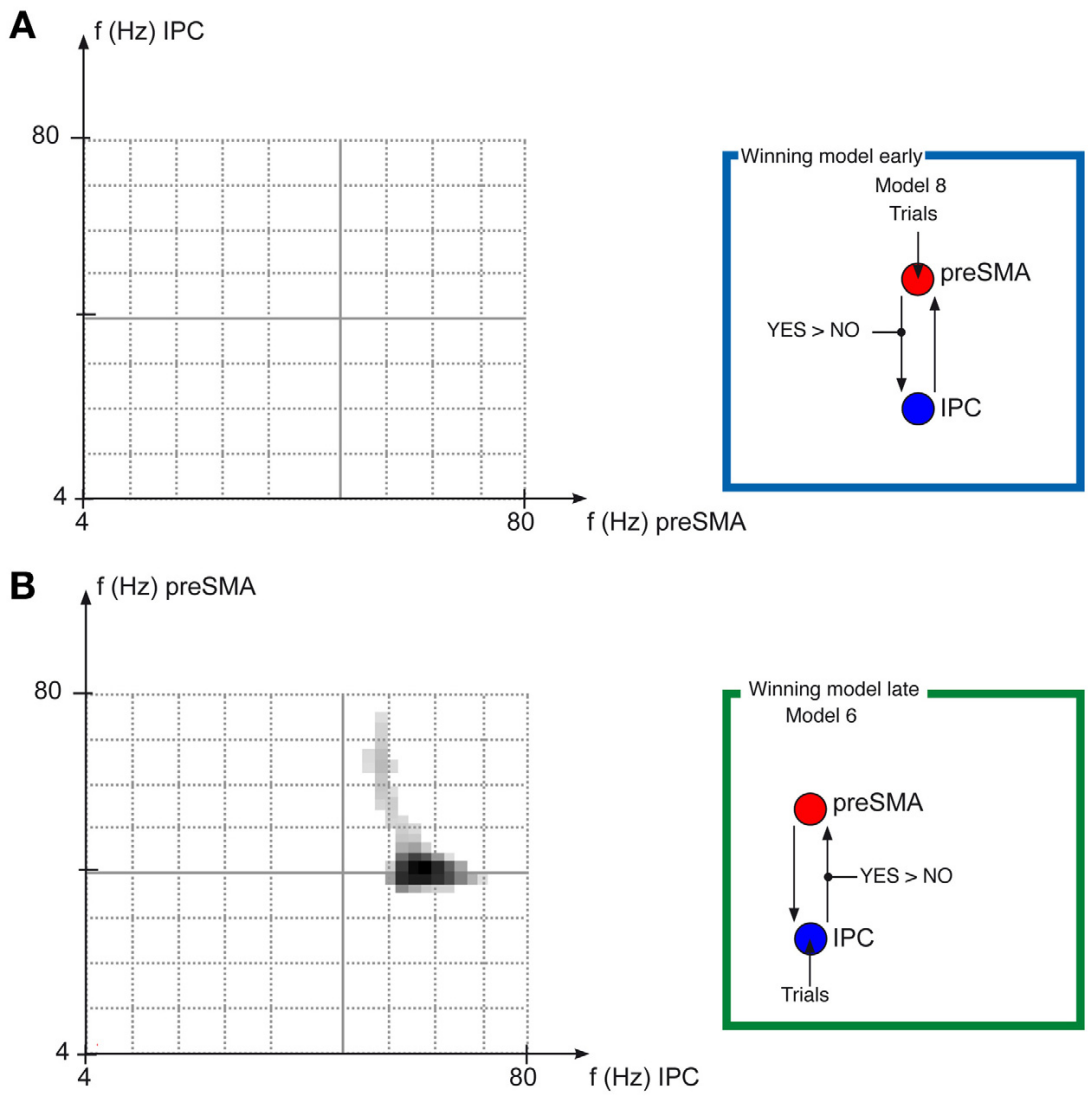
**Frequency-Frequency coupling in winning models**. Results of the DCM analysis frequency-frequency paired *t*-tests. **(A)** Shows the results of the paired *t*-test of Frequency-pairs for the early task phase, testing whether the coupling from preSMA to IPC is significantly (*p* < 0.05 FWE cluster level, based on *p* < 0.05 uncorrected tests of individual frequency pairs) different for AgencyYES compared with AgencyNO trials. **(B)** Shows the results of the paired *t*-test of Frequency-pairs for the late task phase, testing whether the coupling from IPC to preSMA is significantly (*p* < 0.05 FWE cluster level, based on *p* < 0.05 uncorrected tests of individual frequency pairs) different for AgencyYES compared with AgencyNO trials, which is the case in the 50–60 Hz frequency range for IPC, which then increases the power in the frequencies 40–70 Hz increases in preSMA more for AgencyYES compared with AgencyNO.

For the early task phase, where Model 8 was the winning model, the frequency-frequency maps of the couplings from preSMA to IPC revealed no significant (*p* > 0.05 FWE cluster level corrected) (Figure 4A) differences between the AgencyYES and AgencyNO conditions.

For the late task phase, where Model 6 was the winning model, the frequency-frequency maps of the couplings from IPC to preSMA revealed significant differences between the AgencyYES and AgencyNO conditions (*p* < 0.05 FWE cluster level corrected). When the power of frequencies in the range from 50–60 Hz in IPC increased, the power in the frequencies 40–70 Hz increased more in preSMA (Figure 4B) for AgencyYES than for AgencyNO.

## Discussion

In the present study we have investigated SoA and showed that during a simple goal directed computer cursor movement task, a network consisting of two cortical areas that are believed to be involved in SoA display different coupling patterns depending on the state of the movement. Using DCM and BMS, we have shown that the early and late phases of the task are governed by two different processes as revealed by two different dynamics causal models that explain the data best (Model 8 vs. Model 6). Furthermore, we have shown that only in the late phase of the task, the positive SoA, i.e., “yes I am responsible for the action that I have witnessed on the screen in front of me,” is reflected in a change in between-frequency coupling directed from IPC to preSMA in the higher ɣ frequency range. We interpret these findings in the light of the comparator model in such a way that the preSMA processes the intended outcome of the action, and the IPC is used in sensory integration of the visual and proprioceptive feedback. In the case of correspondence of the comparison between the intended outcome of the action and actual feedback, communication between preSMA and IPC is governed by the increased gamma coupling, which thereby becomes essential in order to form SoA because information about a successful comparison has been achieved. The increase in gamma coupling in the direction from IPC to preSMA may suggests that information about the outcome of the corresponding comparison also is fed back to preSMA in order to update intention formation in preSMA as the specific goal of the action now is accomplished.

### Localization, timing, and neural accounts of SoA

These two findings suggest on the one hand that information processing in the neural network underlying the early parts of a goal directed movement is a process that preferentially involves information flow from frontal toward parietal areas, as revealed by the results of the Bayesian model selections. Later, the occurrence of SoA seems to require information about the outcome of the action in order to occur as reflected in coupling with an information flow from parietal to frontal areas. This is consistent with the idea that IPC computes the discrepancy between the intended and actual outcome of the movement performed. Theoretical aspects of SoA imply that a central monitoring system is available in order to estimate congruency or incongruency between motor performance and sensory feedback. This comparator model uses predictions of motor output and actual estimated state of movement (Wolpert et al., 1995; David et al., 2008; Synofzik et al., 2008). This discrepancy is reflected in the larger change in coupling from IPC to preSMA in AgencyYES than that in AgencyNO. This is further reflected in the modulation of oscillatory power in preSMA in the 40–70 Hz range by increases in oscillatory power in IPC in the range from 50–60 Hz.

Although the non-specificity of EEG does not permit a precise localization of the signals, we have used approximate source loci for IPC and preSMA, respectively. In general, EEG source localization cannot be used with the same precision as, for instance, fMRI to determine where specific activity is located in the brain. It is therefore also important to stress that the source localizations performed in this study are of exploratory nature, and that the main effect of SoA reflected as a significant, albeit at very lenient threshold, only indicates that IPC may be related directly to SoA. This may also suggest that the approach to look for neural signatures related to SoA is more likely to be found reflected in the network coupling changes rather than in changes in a single brain region. The IPC has been suggested as important for the conscious intention to move (Sirigu et al., 2004; Desmurget and Sirigu, 2009), and lesions including this area induce an inability to recognize visual feedback of one's own movements (Sirigu et al., 1999). This is well in line with the larger coupling that we observed when participants experienced agency. Farrer and Frith (2002), Farrer et al. (2008) suggested that activity in the angular gyrus, which is part of the IPC, is mainly involved in the rejection of agency. In our study this would be the case when participants realized that the computer rather than they themselves was responsible for the movement. This is not in conflict with our findings, since coupling between two areas says little about the overall activity of the involved areas, and vice versa. We cannot decide from our recordings whether the recording over the preSMA reflected activity in the SMA proper, the preSMA, dorsal premotor cortex, or dorsomedial prefrontal cortex. All areas could potentially be involved, but several recent studies have pointed to the preSMA as the most likely area to be involved in generating the experience of agency (Fried et al., 1991; Moore et al., 2010).

We find it likely that the observed coupling reflects the ongoing introspection of the presence or absence of agency imposed by the experimental setup in this study. This finding is supported by the lack of difference in any of the kinematic parameters regardless of whether or not the participants experienced SoA. Since participants did not change their motor output, it is unlikely that they made their decision of SoA during the motor task. They would appear to depend rather on the subsequent perception and integration of neural signals. Participants have also been reported to be unaware of an external perturbation during drawing of a self-produced line (Fourneret and Jeannerod, 1998). In line with this, Synofzik and co-workers argued that the acknowledgment and judgment of SoA is constructed after the motor task has been performed, as it is based on the interpretation of the failure between predicted vs. performed movement (Synofzik et al., 2013).

We are thus aware that we may have revealed an experimental artifact with little relevance to everyday motor control where agency is taken for granted and only noticed by its (rare) absence (Kuhn et al., 2013). However, this does not change the fact that the observation reflects a genuine neural mechanism related to the conscious experience of agency.

### Frequency ranges

Several studies have provided evidence that conscious perception and attention depend on transient synchronized activity in a distributed network at frequencies around 30–60 Hz (Rodriguez et al., 1999; Engel et al., 2001; Palva et al., 2005; Melloni et al., 2007; Siegel et al., 2012).

The increased ɣ-coupling that we observe seems to be a genuine finding specifically related to participants' perception of agency, because there are no attentional differences associated with the two subjective states imposed by the experimental setup. Furthermore, we base this statement on the fact that there were no behavioral differences with respect to movement time and reaction time in the two different subjective states, which could have indicated different attentional load. One study has revealed that ɣ-power in cingulate motor areas correlates with performance in a task where participants have to monitor their internal attentional state (Yamagishi and Anderson, 2013). However, this finding was not associated with coupling changes. Neural signatures of attentional mechanisms are indeed also displayed as top down modulation of ɣ-band coupling (Siegel et al., 2012). However, our findings do differ [from what?] in showing increased ɣ-coupling in a specific network, with a specific directionality of the coupling. It is not as a top-down controlled mechanism, but rather as a modulation of the bottom-up information giving the flow direction, i.e., from IPC to preSMA, which is in contrast to the more generalized long-distance synchrony observed in the previous studies (Melloni et al., 2007) reflecting a top-down attentional modulation (Siegel et al., 2012). The more generalized long-distance synchrony in the ɣ-band is probably linked to non-specific conscious awareness or attentional top down mechanisms rather than to processing of specific features of the perceived sensorimotor information. It is likely that conscious detection of other specific sensory features will reveal a specific coupling in different relevant local circuitries similar to what we have seen here.

As seen in Supplementary Figure S1, the DCMs models the observed time-frequency content of the two source regions quite well. Importantly it is also evident that the two regions display quite different tempero-frequency dynamics in all participants, suggesting, that coupling is not due to common noise signals in the two regions.

### Limitations to our study

Unfortunately it was not possible to have a single deviation degree that gave rise to a 50/50 distribution of AgencyYES and AgencyNO responses in all participants, and we were therefore forced to collapse all trials across different deviation angles. This will naturally give rise to more small angle (i.e., +/−1°) deviations in the AgencyYES condition and large angle (i.e., +/−6°) deviations in the AgencyNO conditions. However, we do not believe that the difference in coupling between AgencyYES and AgencyNO is a reflection of purely larger visual deviations (+/−1 vs. 6°). As the deviation is initiated shortly after the movement starts, we would also have seen a similar difference in coupling for the early task phase where the visual deviation also is present.

DCM implies causality at the structural level, which means that causality is inferred by how the state equations of the DCM are coupled, and not by temporal precedence of activity in one area and then later in another area. If there is, as we suggest, a different causal relation between the investigated regions in the early and late task phases, it would not have been possible to integrate that into one large DCM covering the full time window of the task. This means that it would not be possible to integrate the dynamics of the whole task into a single model, if one expects that the directional communication changes throughout the task. Therefore, the approach with the split of the data into an early and a late task phase was employed.

## Conclusion

In conclusion our observations are consistent with the idea that the sense of agency is mainly determined *post-hoc* based on a comparison between the sensory consequences of the movement and the original intention, rather than the ongoing experience during the movement (Kawato and Wolpert, 1998). The sudden absence of agency that we may experience when our interaction with the environment is suddenly altered (defective computer mouse or defective steering in a car) may then be signaled by the absence of high ɣ coupled activity in IPC and preSMA, when comparison of sensory feedback and motor plan reveals that the desired target was not obtained. This idea requires further testing.

## Author contributions

Anina Ritterband-Rosenbaum, Jens B. Nielsen, and Mark S. Christensen designed the experiment and Anina Ritterband-Rosenbaum carried out the data collection. Anina Ritterband-Rosenbaum and Mark S. Christensen analyzed the data, and Anina Ritterband-Rosenbaum, Mark S. Christensen, and Jens B. Nielsen wrote the manuscript.

### Conflict of interest statement

The authors declare that the research was conducted in the absence of any commercial or financial relationships that could be construed as a potential conflict of interest.

## Supplementary material

Supplementary Figure S1 **Time-frequency plots observed and predicted from DCM**. Comparison of time-frequency plots of the observed two source regions (preSMA and IPC) for the **(A)** early and **(B)** late task phases for AgencyYES and AgencyNO trials with the predictions made by the DCMs (model 8 early and model 6 late). Notice for one participant (shaded) no dynamics were predicted by any of the 9 DCMs (here only model 6 is displayed) for the late task phase.
