# Supplementary figures and images for: Sense of agency is related to gamma band coupling in an inferior parietal-preSMA circuitry
Source: Front Hum Neurosci. 2014 Jul 16;8:510. doi: 10.3389/fnhum.2014.00510 (PMC4100320; doi:10.3389/fnhum.2014.00510)

# A - Early task phase

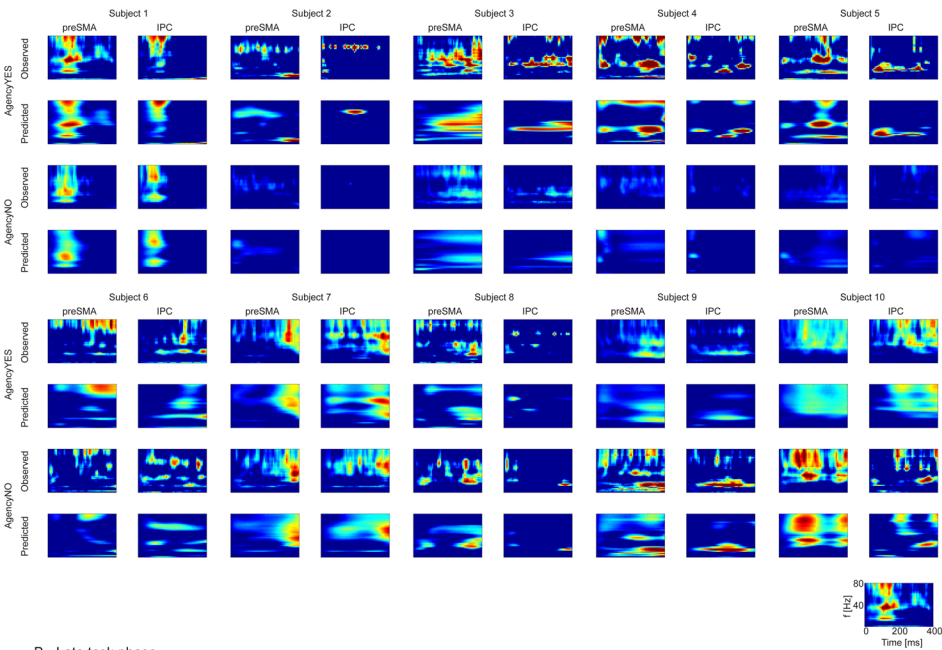

# B - Late task phase

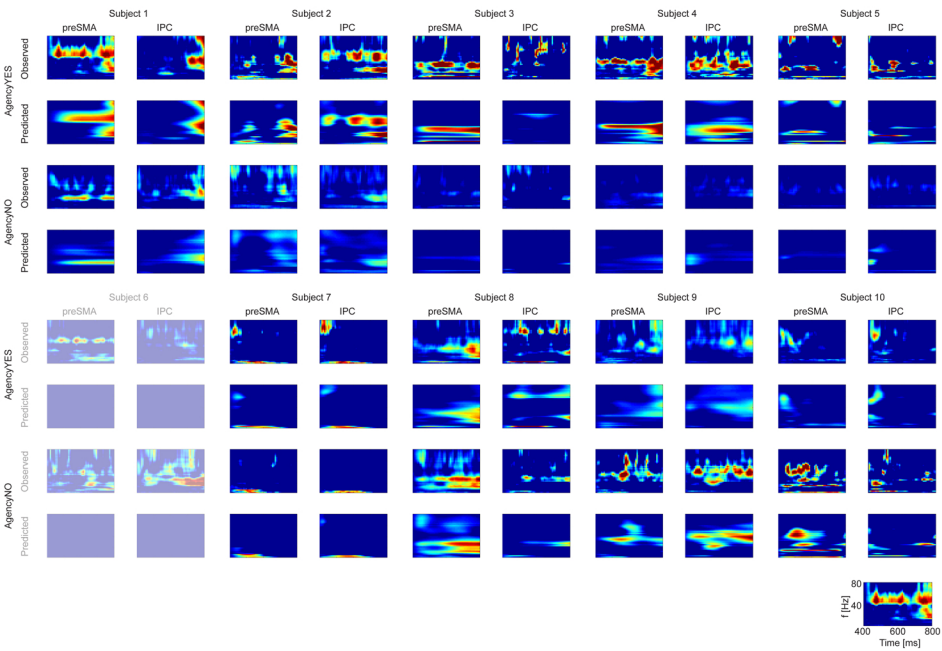

Supplement: Supplementary Figure S1 — Time-frequency plots observed and predicted from DCM. Comparison of time-frequency plots of the observed two source regions (preSMA and IPC) for the (A) early and (B) late task phases for AgencyYES and AgencyNO trials with the predictions made by the DCMs (model 8 early and model 6 late). Notice for one participant (shaded) no dynamics were predicted by any of the 9 DCMs (here only model 6 is displayed) for the late task phase. [file Presentation1.PDF]
